# Arrhythmogenic Ventricular Remodeling by Next-Generation Bruton’s Tyrosine Kinase Inhibitor Acalabrutinib

**DOI:** 10.3390/ijms25116207

**Published:** 2024-06-05

**Authors:** Yanan Zhao, Praloy Chakraborty, Julianna Tomassetti, Tasnia Subha, Stéphane Massé, Paaladinesh Thavendiranathan, Filio Billia, Patrick F. H. Lai, Husam Abdel-Qadir, Kumaraswamy Nanthakumar

**Affiliations:** 1 Toronto General Hospital Research Institute, University Health Network, Toronto, ON M5G 2M1, Canada; ynzhao17@mails.jlu.edu.cn (Y.Z.); praloy.chakraborty@uhn.ca (P.C.); juliannatomassetti101@gmail.com (J.T.); tasniasubha@outlook.com (T.S.); stephane.masse@uhn.ca (S.M.); dinesh.thavendiranathan@uhn.ca (P.T.); phyllis.billia@uhn.ca (F.B.); patrick.lai@uhn.ca (P.F.H.L.); husam.abdel-qadir@uhn.ca (H.A.-Q.); 2 Ted Rogers Centre for Heart Research, Toronto, ON M5G 1M1, Canada; 3 Women’s College Hospital, Toronto, ON M5S 1B2, Canada

**Keywords:** ibrutinib, acalabrutinib, action potential, calcium cycling, ventricular arrhythmia, electrical remodeling

## Abstract

Cardiac arrhythmias remain a significant concern with Ibrutinib (IBR), a first-generation Bruton’s tyrosine kinase inhibitor (BTKi). Acalabrutinib (ABR), a next-generation BTKi, is associated with reduced atrial arrhythmia events. However, the role of ABR in ventricular arrhythmia (VA) has not been adequately evaluated. Our study aimed to investigate VA vulnerability and ventricular electrophysiology following chronic ABR therapy in male Sprague–Dawley rats utilizing epicardial optical mapping for ventricular voltage and Ca^2+^ dynamics and VA induction by electrical stimulation in ex-vivo perfused hearts. Ventricular tissues were snap-frozen for protein analysis for sarcoplasmic Ca^2+^ and metabolic regulatory proteins. The results show that both ABR and IBR treatments increased VA vulnerability, with ABR showing higher VA regularity index (RI). IBR, but not ABR, is associated with the abbreviation of action potential duration (APD) and APD alternans. Both IBR and ABR increased diastolic Ca^2+^ leak and Ca^2+^ alternans, reduced conduction velocity (CV), and increased CV dispersion. Decreased SERCA2a expression and AMPK phosphorylation were observed with both treatments. Our results suggest that ABR treatment also increases the risk of VA by inducing proarrhythmic changes in Ca^2+^ signaling and membrane electrophysiology, as seen with IBR. However, the different impacts of these two BTKi on ventricular electrophysiology may contribute to differences in VA vulnerability and distinct VA characteristics.

## 1. Introduction

The introduction of Bruton’s tyrosine kinase inhibitors (BTKi) is an important therapeutic advance in the management of B-cell lymphocyte malignancies [1]. This targeted therapeutic strategy avoids the cytotoxic side effects of conventional cancer chemotherapeutics. However, Ibrutinib (IBR)-associated cardiotoxicity is a significant concern, with cardiac arrhythmias being the most common cardiotoxic side effect [2,3]. Although atrial fibrillation (AF) is the most common cardiotoxicity associated with IBR, often prompting treatment cessation [3,4], ventricular arrhythmia (VA) also presents a substantial burden and is potentially fatal [5,6]. Off-target inhibition of other cellular kinases by IBR, a covalent BTKi, is thought to be responsible for cardiovascular side effects, including arrhythmias [7]. Acalabrutinib (ABR) is a next-generation selective BTKi with reduced off-target kinase inhibition [8]. In a head-to-head comparison of clinical efficacy in chronic lymphocytic leukemia, ABR was found to be non-inferior to IBR in progression-free survival, with a significantly lower incidence of AF [9]. A higher selectivity for BTK inhibition with a lesser degree of inhibition of off-target C-terminal Src Kinase (CSK), PI3K-Akt signaling, and Ca^2+^/Calmodulin-dependent protein kinase II (CaMKII) may explain a lower incidence of AF with ABR [7,10,11,12].

Despite the small number of investigations that have drawn attention to VAs associated with ABR [13,14], its impact on VA remains inadequately assessed and the underlying mechanisms of ABR-induced VAs remain unclear. Our group has demonstrated that acute as well as chronic IBR treatments are associated with the aberration of membrane voltage and intracellular calcium (Ca^2+^) dynamics in the heart, with a subsequent increase in VA vulnerability [15,16]. We hypothesized that ABR and IBR have distinct effects on membrane electrophysiology and calcium signaling, resulting in different levels of VA vulnerability and characteristics. We evaluated the effects of chronic ABR treatment on ventricular electrophysiology, Ca^2+^ dynamics, and arrhythmia susceptibility in a rodent model. We also compared the arrhythmogenic alteration in ventricular electrophysiology, VA susceptibility, VA characteristics, Ca^2+^ handling, and metabolic regulatory proteins between IBR and ABR treatments. This comparison may shed light on the differing clinical outcomes observed between IBR and ABR.

## 2. Results

### 2.1. Effects of IBR and ABR Treatment on VA Vulnerability and Characteristics

**Figure 1:**
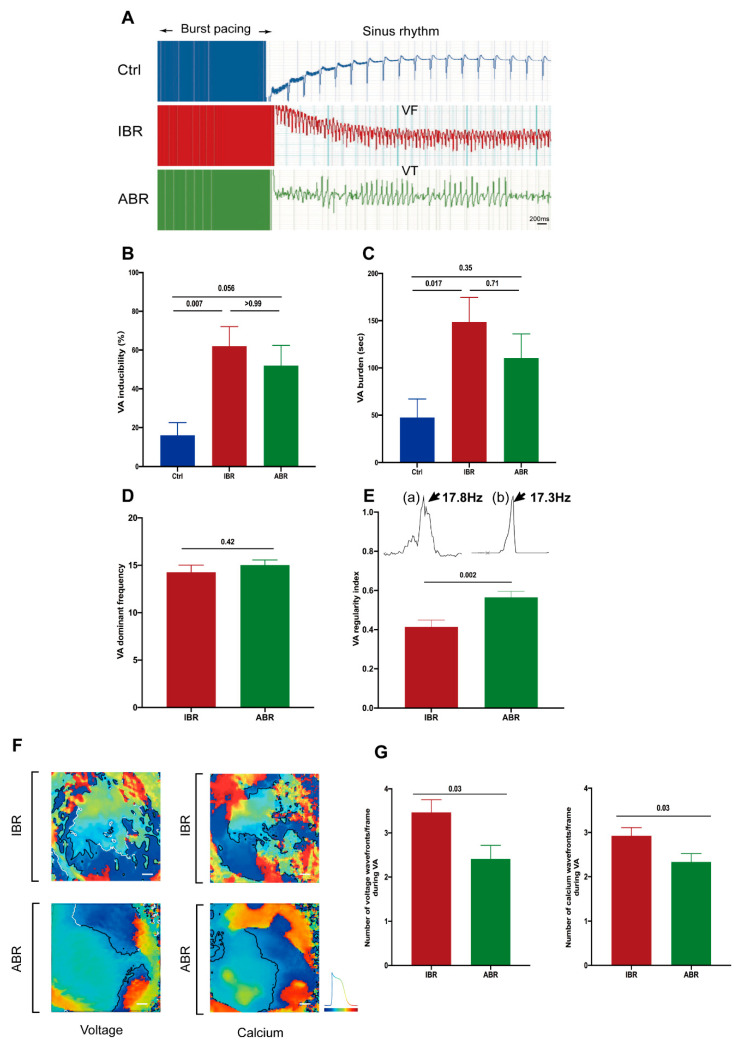
Effects of Ibrutinib (IBR) and Acalabrutinib (ABR) on ventricular arrhythmia (VA) vulnerability and characteristics. (**A**) Representative pseudo-electrogram (pseudo-ECGs) after VA inductions in the heart of control (Ctrl) and IBR- and ABR-treated rats. (**B**) VA inducibility and (**C**) VA burden in the ABR-treated group were higher compared to control but lower than those in the IBR-treated group (*n* = 10 in each group; *p*-value: Kruskal–Wallis test). (**D**) Dominant Frequencies (DF) of induced VA were similar in the IBR- and ABR-treated groups (*n* = 7 in each group; *p*-value: *t*-test). (**E**) The Regularity Index (RI) of induced VA was higher in the ABR-treated group compared to the IBR-treated group. Representative (a) IBR and (b) ABR frequency distribution during VA (*n* = 7 in each group; *p*-value: *t*-test). (**F**) Illustrative voltage and calcium phase mapping during VA in the hearts from IBR- and ABR-treated rats. Scale bar: 20 mm. (**G**) Number of membrane voltage and calcium wavefronts per frame during early (10 s) and late (10 s) VAs in IBR and ABR groups (*n* = 7–8 in each group; *p*-value: Kruskal–Wallis test or *t*-test).

The effect of burst pacing is shown in Figure 1A. IBR treatment markedly increased VA vulnerability (62.0 ± 10.1% in IBR vs. 16.0 ± 6.5% in control; *p* = 0.007) and VA burden (148.7 ± 30.0 s in IBR vs. 47.6 ± 19.4 s in control; *p* = 0.017), compared to control hearts. The trends for VA vulnerability and VA burden with ABR treatment were higher than those of the control (52.0 ± 10.4% in ABR vs. 16.0 ± 6.5% in control for VA vulnerability; 110.5 ± 25.4 s in ABR vs. 47.6 ± 19.4 s in control for VA burden) but lower than seen with IBR treatment (52.0 ± 10.4% in ABR vs. 62.0 ± 10.1% in IBR for VA vulnerability; 110.5 ± 25.4 s in ABR vs. 148.7 ± 30.0 s IBR for VA burden). Although the ABR-treated hearts showed a tendency to higher VA vulnerability, as compared to control (*p* = 0.056), this did not achieve statistical significance. Similarly, statistical analysis did not demonstrate a significant difference in VA vulnerability between ABR-treated hearts and IBR-treated hearts (*p* > 0.99), and the VA burden was not statistically significant in ABR-treated hearts as compared to control (*p* = 0.35), or as compared to IBR-treated hearts (*p* = 0.71) (Figure 1B,C). Dominant frequency (DF) during induced VA was similar following IBR and ABR treatments (15.02 ± 0.55 Hz in ABR vs. 14.27 ± 0.74 Hz in IBR for VA; *p* = 0.42) (Figure 1D). However, ABR treatment was associated with a higher regularity index (RI) of VA compared to VAs in IBR-treated hearts (0.56 ± 0.03 in ABR vs. 0.41 ± 0.03 in IBR; *p* = 0.002) (Figure 1E), suggesting a more organized VA pattern with ABR treatment. Figure 1F shows voltage and calcium phase maps of the epicardial surface of rat hearts in the IBR and ABR groups, suggesting that hearts from IBR-treated rats exhibit a more spatiotemporal disorganized phase map during VA than those from ABR-treated rats (Videos S1 and S2). The number of voltage and calcium wavefronts for the first and last ten seconds during VA were measured. Both the mean numbers of voltage (3.47 ± 0.29/frame vs. 2.41 ± 0.31/frame in ABR, *p* = 0.03) and Ca^2+^ (2.93 ± 0.18/frame vs. 2.33 ± 0.19/frame in ABR, *p* = 0.03) wavefronts were higher in the IBR group (Figure 1G). The pseudo-electrogram (pseudo-ECG) also showed results consistent with the above observation; the VAs in the ABR group were primarily monomorphic, and this was significantly different from the predominant polymorphic VT of the IBR group (*p* = 0.015) (Figure S1).

### 2.2. Effects of IBR and ABR on Ventricular Electrophysiology

**Figure 2:**
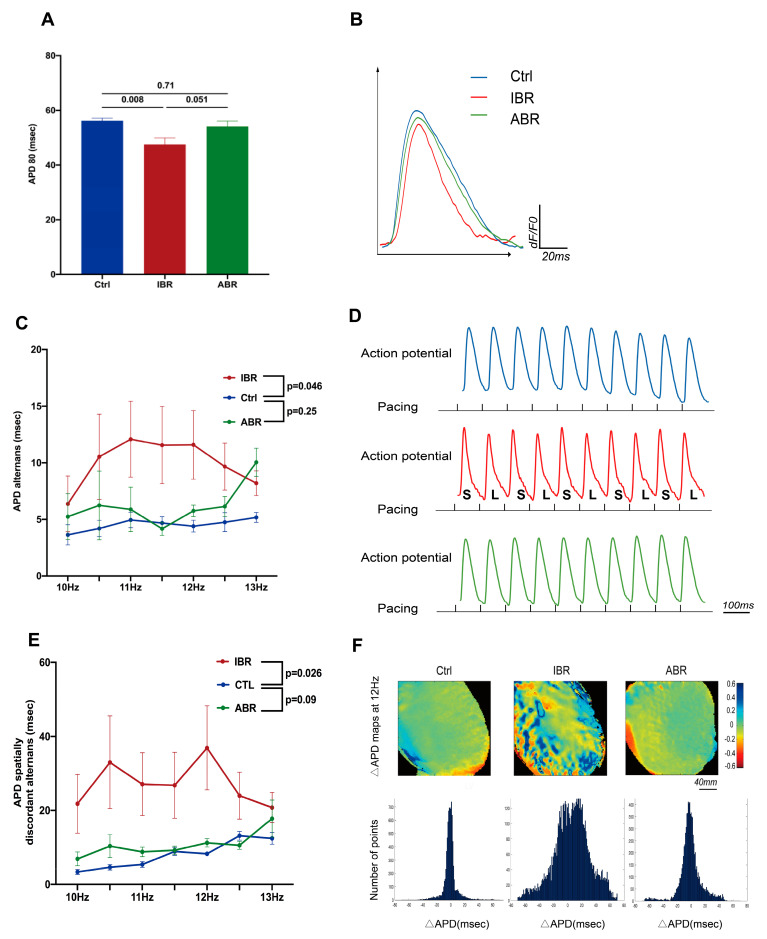

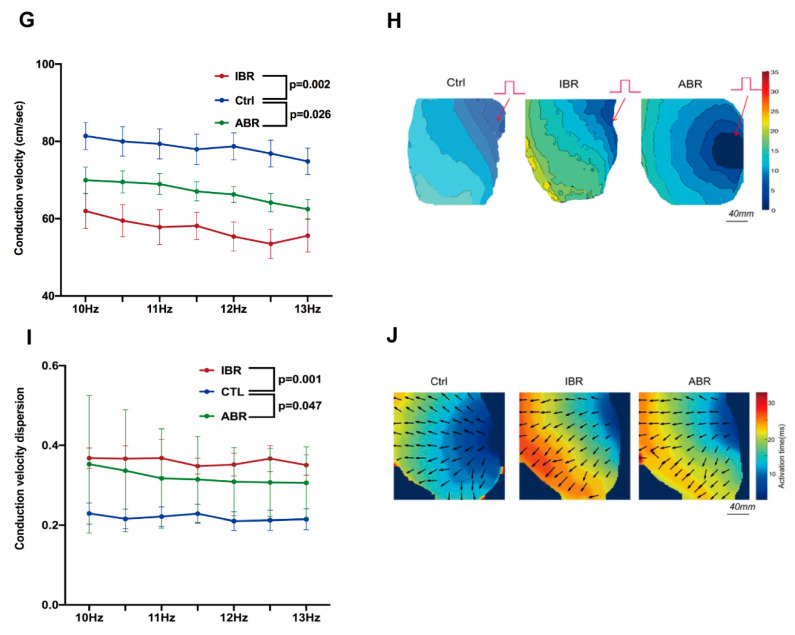
Effects of IBR and ABR on ventricular electrophysiology. (**A**) IBR, but not ABR, treatment was associated with reduced action potential duration (APD) at 80% repolarization (APD_80_) (*n* = 10 at each group; *p*-value: *t*-test). (**B**) Representative traces of action potential at 10 Hz. (**C**) APD alternans was higher with IBR therapy, but not with ABR therapy. (**D**) Representative tracings with increased APD alternans from an IBR-treated heart and reduced APD alternans from Ctrl and ABR hearts at a pacing rate of 11.0 Hz. (**E**) IBR increased APD spatial discordance alternans. (**F**) Representative maps showing dispersion of APD_80_, with color code, at 12 Hz. Histograms showing the distributions of ΔAPD across the LV epicardial mapping area, 12 Hz pacing (*x*-axis: ΔAPD [ms]; *y*-axis: the number of points from the ΔAPD map) (*n* = 8–10; *p*-value: two-way repeated ANOVA). (**G**)Epicardial conduction velocity (CV) was lower with IBR and ABR treatments. (**H**) Representative successive isochronal conduction maps with 10 Hz pacing. Red arrows show the pacing location. (**I**) CV dispersion was higher with IBR and ABR therapies. (**J**) Representative color maps showing CV dispersion in different groups during 10 Hz pacing. The arrows indicates the conduction direction. (**C**–**J**: *n* = 8–10 in each group; *p*-value: two-way repeated ANOVA).

As shown in Figure 2, IBR treatment was associated with the shortening of the durations, from time 0 to 80%, relative to repolarization of action potential duration (APD_80_) (*p* = 0.008) (Figure 2A,B), potentiated action potential duration (APD) alternans (*p* = 0.046), (Figure 2C,D) and significant APD spatially discordant alternans (*p* = 0.026) (Figure 2E,F). In contrast, ABR did not change the APD_80_ (*p* = 0.71), APD alternans (*p* = 0.25), or APD spatially discordant alternans (*p* = 0.09), compared to the control. Interestingly, both IBR and ABR treatments were associated with a significant reduction of conduction velocity (CV) (*p* = 0.002 for IBR and *p* = 0.026 for ABR) (Figure 2G,H) and an increase in CV dispersion (*p* = 0.001 for IBR and *p* = 0.047 for ABR) (Figure 2I,J), compared to controls.

### 2.3. Effects of IBR and ABR on Ventricular Calcium Dynamics

**Figure 3:**
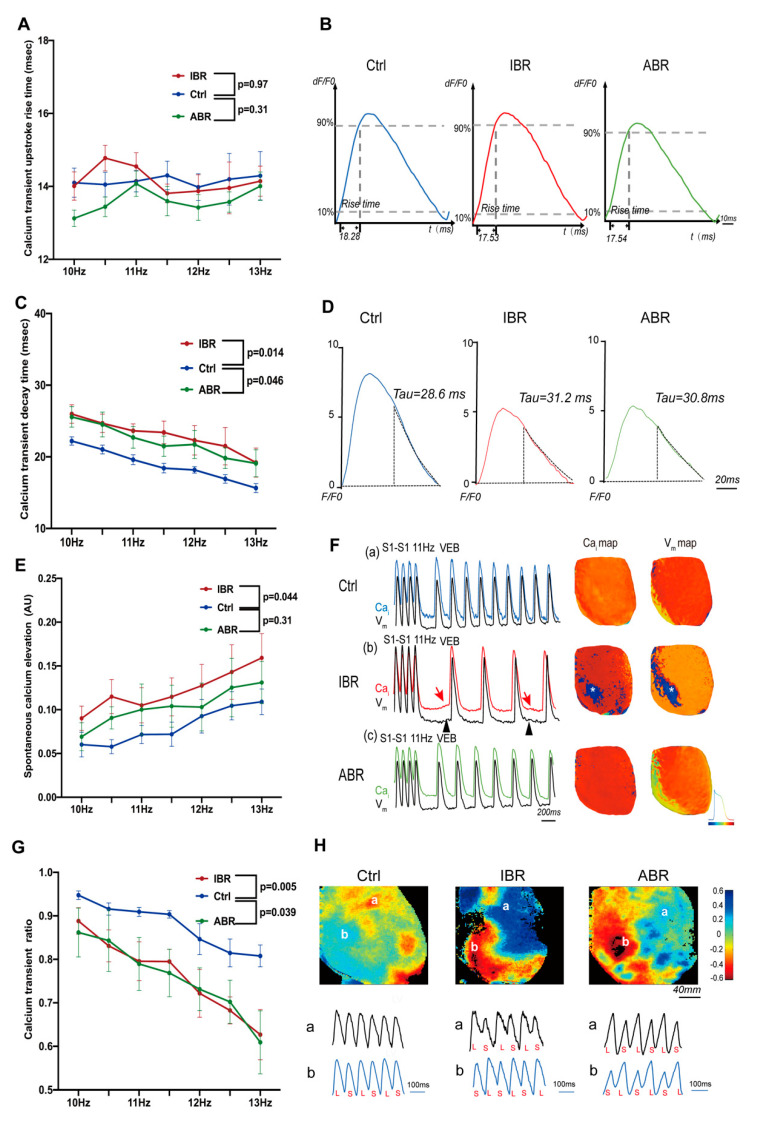
Effects of IBR and ABR on ventricular calcium cycling. (**A**) There were no differences in calcium transient (CaT) upstroke rise with IBR and ABR treatments. (**B**) Representative traces of CaT upstroke rise at 10 Hz. (**C**) The decay time constant of CaT was larger with IBR and ABR treatments. (**D**) Representative CaTs at 10 Hz. The decay portion of the CaT is marked as a black curve. (**E**) IBR, but not ABR, treatment was associated with significantly a greater spontaneous calcium elevation (SCaE). (**F**) Representative traces of SCaE during 11 Hz pacing. (a–c) Indications of optical tracing and color maps of intracellular calcium (Ca_i_) and membrane voltage (V_m_), respectively. (b) SCaE (red arrows) and delayed after–depolarization (DAD) (black arrowhead) were elicited at the cessation of rapid pacing in the heart of IBR–treated rats. Optical images were captured from the sites labeled by asterisks in Cai and V_m_ maps. (**G**) More calcium alternans is observed with IBR and ABR treatments. (**H**) Color maps of calcium alternans and corresponding CaT traces at 12 Hz, a,b represent different heart regions of interest. (**A**–**H**: *n* = 9–10 in each group; *p*–value: two–way repeated ANOVA). VEB—ventricular escape beat.

The effects of IBR and ABR treatments are shown in Figure 3. Neither IBR nor ABR treatment demonstrated a change in calcium transient (CaT) rise time compared to the control (*p* = 0.97 for IBR and *p* = 0.31 for ABR) (Figure 3A,B). However, CaT decay time (Tau value) was prolonged by IBR (*p* = 0.014) and ABR (*p* = 0.046), suggesting impaired diastolic Ca^2+^ clearance by both agents (Figure 3C,D). Spontaneous calcium elevation (SCaE), a marker of diastolic Ca^2+^ leak, was induced by IBR (*p* = 0.044), but not ABR (*p* = 0.31), treatment, and SCaE-induced spontaneous diastolic depolarization in IBR treated left ventricles (Figure 3E,F). CaT amplitude ratio was significantly reduced in IBR- (*p* = 0.005) and ABR-treated (*p* = 0.039) hearts, indicating potentiation of CaT alternans by these agents (Figure 3G,H).

### 2.4. Effects of IBR and ABR on Calcium-Handling and Metabolic Regulatory Proteins

**Figure 4:**
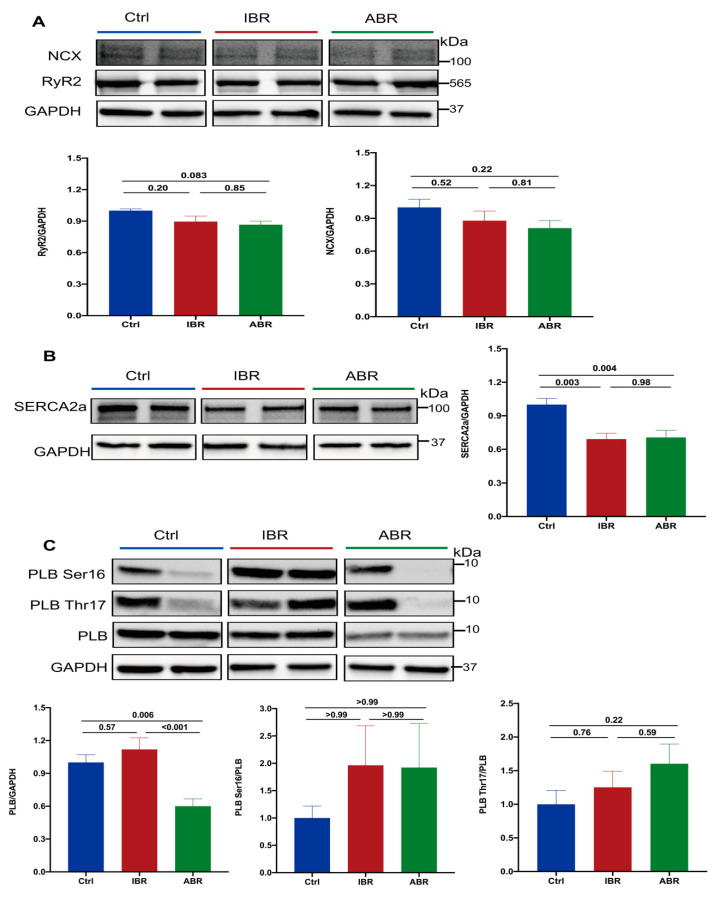

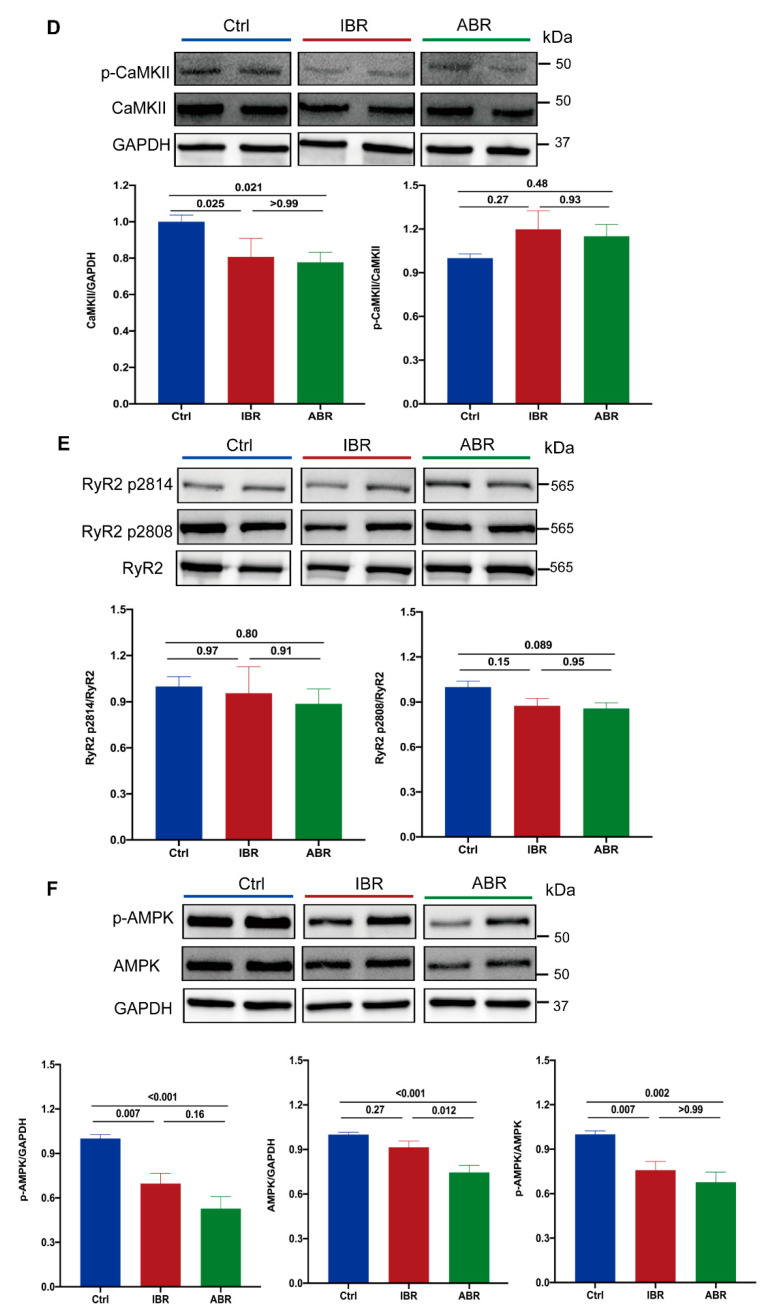
Effects of IBR and ABR on calcium-handling and metabolic regulatory proteins. Representative gel blots and normalized expression of (**A**) Na^+^-Ca^2+^ exchanger (NCX) and total RyR2; *n* = 6–9 in each. (**B**) Sarcoplasmic reticulum Ca^2+^-ATPase 2a (SERCA2a); *n* = 9–10 in each. (**C**)Phospholamban (PLB), and phosphorylation of PLB at Threonine-17 and Serine-16. (**D**) Calcium/calmodulin-dependent kinase, type II (CaMKII); phosphorylation of CaMKII at Thr 286 or 287. **C**,**D**: *n* = 9 in each. (**E**) Ryanodine receptor type 2 (RyR2) and phosphorylation of RyR2 at Ser2814 and Ser2808; *n* = 6 in Ctrl and *n* = 8 in IBR and ABR groups. (**F**) 5′-adenosine monophosphate-activated protein kinase (AMPK), phosphorylation of AMPK at Thr 172; *n* = 9 in each, between IBR, ABR, and Ctrl groups. The *p* value for (**A**–**F**): one-way ANOVA or Kruskal–Wallis test. GAPDH—glyceraldehyde 3-phosphate dehydrogenase.

To understand the mechanisms of abnormal calcium and voltage dynamics, we evaluate the abundance and phosphorylation of sarcoplasmic calcium-handling proteins and the metabolic regulator 5′-adenosine monophosphate-activated protein kinase (AMPK). There were no changes in the protein abundance of ryanodine receptor type 2 (RyR2) or Na^+^-Ca^2+^ exchanger (NCX) with either IBR or ABR (Figure 4A). However, sarcoplasmic reticulum Ca^2+^-ATPase 2a (SERCA2a) protein abundance was reduced by both agents (*p* = 0.003 for IBR and *p* = 0.004 for ABR) (Figure 4B). Protein abundance of phospholamban (PLB) was reduced by ABR (*p* = 0.006) and calcium/calmodulin-dependent kinase, type II (CaMKII) was reduced by IBR (*p* = 0.025) and ABR (*p* = 0.021); the degree of phosphorylation of these molecules was not altered by either of these agents (Figure 4C,D). Similarly, Ser 2814 and Ser 2808 phosphorylation of RyR2 were not changed by IBR (*p* = 0.97 for ser 2814; *p* = 0.15 for ser 2808) or ABR (*p* = 0.80 for ser 2814; *p* = 0.089 for ser 2808) (Figure 4E). IBR decreased AMPK phosphorylation (*p* = 0.007), whereas ABR reduced the protein ex abundance of AMPK (*p* < 0.001) and phosphorylated AMPK (*p* < 0.001). Hence, the *p*-AMPK/AMPK ratio was reduced by both IBR (*p* = 0.007) and ABR (*p* = 0.002) (Figure 4F).

We further explored the impacts of ABR on the levels of C-terminal Src kinase (CSK) and the phosphoinositol-3 kinase (PI3K)-Akt signaling pathway. Our analysis demonstrated no significant differences in CSK expression (*p* = 0.98; Figure S2A) or PI3K expression (*p* = 0.59; Figure S2B) between the control and ABR-treated groups. Additionally, there were no significant differences in Akt phosphorylation between the control and ABR groups (*p* = 0.70; Figure S2B).

## 3. Discussion

In our study, IBR treatment was associated with increased VA vulnerability and VA burden. The trend of VA vulnerability in ABR-treated hearts was higher than in controls but lower than in IBR-treated hearts, although the results did not reach statistical significance. Notably, the ABR-induced VAs were more spatially organized, compared to those with IBR. IBR treatment resulted in abnormalities in ventricular repolarization, as indicated by abbreviated APD and increased APD dispersion, as well as conduction abnormalities characterized by decreased CV and higher CV dispersion. In contrast, ABR treatment was associated only with abnormal ventricular conduction properties, including reduced ventricular CV and higher CV dispersion, albeit with a lower magnitude of CV dispersion abnormalities compared to IBR treatment. Both IBR and ABR treatments were associated with pro-arrhythmic calcium cycling abnormalities, including diastolic Ca^2+^ clearance and Ca^2+^ alternans.

### 3.1. Acalabrutinib and Ventricular Arrhythmia

Abnormal ventricular electrophysiology and increased VA risk caused by IBR have been demonstrated by experimental and clinical studies [6,17,18]. As a more selective BTKi, ABR is supposed to be less arrhythmogenic. However, there are reports of symptomatic VAs associated with next-generation BTKi [19]. Additionally, a follow-up study has demonstrated that while the VA risk with ABR is higher than that of the control, it remains lower than rates observed with IBR therapy [13]. Our study also suggests that although ABR treatment is associated with a higher risk of inducible VA and VA burden, the risk is lower compared to that with IBR. Furthermore, our study showed that induced VA in the heart of IBR-treated rats exhibited greater spatial disorganization than seen in hearts from the ABR group, making them more likely to cause insurmountable hemodynamic instability [20]. This finding aligns with the existing published literature on the characteristics of VA in patients treated with IBR and ABR [13,21,22,23]. The different impacts of IBR and ABR on ventricular membrane voltage, as demonstrated in our study, may underlie the varying vulnerability to VA and the distinct VA characteristics observed between these two agents [9].

### 3.2. Acalabrutinib and Ventricular Membrane Voltage

In our study, IBR, but not ABR, was associated with the abbreviation of APD, increased beat-to-beat APD alternans and seriously spatially discordant APD alternans. An abbreviated APD correlates with a shortened ventricular refractory period. On the other hand, spatial dispersion of APD (APD alternans) is known to be arrhythmogenic, and the risk is more with spatially discordant APD alternans [24,25]. The spatial dispersion of APD, especially the plateau phase, produces dynamic areas of unidirectional conduction block, facilitating the formation of functional reentry substrate [26,27,28]. On top of that, the diastolic Ca^2+^ overload generates the early after-depolarizations which trigger the induction of circus movement reentry and polymorphic ventricular tachycardia (PVT)/VF [26]. Depolarization heterogeneity from increased CV dispersion potentiates the functional reentry substrate of VA’s incremental effect in creating the lines of block [29]. Both decreased CV and the reduced refractory period from abbreviated APD further promote the propagation of the reentrant PVT/VF by reducing the wavelength of the circuit [27].

ABR treatment was associated with impaired conduction properties, including decreased CV and increased CV dispersion without any repolarization abnormalities, compared to IBR. Isolated regional CV heterogeneity produces areas of functional unidirectional block, especially in presence of a premature ventricular beat [30]. The unidirectional block created by conduction heterogeneity, along with slow conduction, creates functional re-entry circuits for VA, leading to the generation of monomorphic ventricular tachycardia (VT) [29,30]. The reentry circuits, in this setting, are commonly stable and fixed, leading to the generation of more organized VA [30,31].

### 3.3. Acalabrutinib and Ventricular Calcium Cycling

Both IBR and ABR therapies resulted in impaired diastolic Ca^2+^ clearance, as evidenced by an increase in diastolic Ca^2+^ decay time. Additionally, IBR treatment induced SCaE, which reflects a diastolic Ca^2+^ leak. These findings suggest that both IBR and ABR may elevate intracellular Ca^2+^ levels during diastole (↑ [Ca^2+^]_i_). Abnormal ventricular Ca^2+^ signaling was previously reported following administration of IBR [15,16]. In the presence of higher diastolic [Ca^2+^]_i_, excess Ca^2+^ is extruded out of the cell in the exchange due to the intrusion of 3 sodium ions (Na^+^) by electrogenic sodium-calcium exchange protein (NCX), leading to diastolic depolarization [32]. The diastolic depolarization subsequently produces a premature ventricular beat, which acts as a trigger for VA. Higher intracellular Ca^2+^ may also contribute to IBR- and ABR-induced reduction in CV [33]. In our study, CaT alternans was potentiated by both IBR and ABR. CaT alternans, the beat-to-beat alteration of CaT amplitude, is another manifestation of malfunction in sarcoplasmic Ca^2+^ handling. Enhanced CaT alternans is linked to ventricular arrhythmogenesis. Intracellular Ca^2+^ is shown to modulate APD by electrogenic feedback, and increased CaT alternans is associated with arrhythmogenic APD alternans [34]. To understand the mechanisms of aberrant Ca^2+^ signaling, we evaluated the expression and phosphorylation status of important Ca^2+^ handling proteins. Increased phosphorylation and activity of RyR2 is an important mechanism of abnormal Ca^2+^ signaling in a variety of cardiac conditions [32]. IBR, as well as ABR, did not modify the phosphorylation of Ser2808 and Ser2814 of RyR2 or CaMKII, ruling out CaMKII activation by both agents [32]. SERCA2a is largely responsible for sarcoplasmic Ca^2+^ uptake during diastole [32]. PLB, when associated with SERCA2a, inhibits SERCA2a activity. However, phosphorylation of PLB by protein kinase A (PKA) and CaMKII promotes dissociation of PLB from SERCA2a and increases SERCA2a activity. PLB phosphorylation was also not modified by IBR or ABR. Interestingly, SERCA2a expression was reduced by IBR and ABR. Importantly, reduced SERCA2a expression is associated with reduced SR Ca^2+^ load, diastolic Ca^2+^ overload, Ca^2+^ leak, and CaT alternans [35]. Overexpression of SERCA2a by genetic manipulation has been reported to improve cytosolic Ca^2+^ dynamics and attenuate VA [35,36]. Hence, BTKi-induced reduction in SERCA2a expression may be responsible for proarrhythmic remodeling of intracellular Ca^2+^ signaling in our study.

### 3.4. Acalabrutinib and Cardiac Metabolic Regulation

In our study, both steady-state protein levels and phosphorylation of AMPK were reduced by ABR, whereas IBR reduced AMPK phosphorylation. This finding aligns with observations in the context of the tyrosine kinase inhibitor sunitinib [37], suggesting a plausible class effect of constrained AMPK activation attributable to these two covalent BTKis [9]. AMPK is an important cellular-stress-sensing molecule that is activated by phosphorylation during metabolic challenges and helps to maintain cellular energy homeostasis by promoting catabolic pathways and improving mitochondrial biogenesis and function [38]. Other than metabolic control, AMPK also plays an important role in sarcoplasmic Ca^2+^ handling and membrane ion homeostasis, and reduced AMPK activity is linked to atrial and ventricular arrhythmias [38,39]. The reduced expression and phosphorylation of AMPK are associated with decreased AMPK activity and abnormal Ca^2+^ dynamics by BTKi molecules in our study, which may have resulted from decreased AMPK activity. The AMPK reserve is diminished with aging, and further AMPK inhibition is reported to exacerbate the aging-associated impairment of diastolic Ca^2+^ clearance [40]. Similarly to our study, Turdi et al. also demonstrated that decreased AMPK function is primarily associated with reduced SERCA2a expression without any modification of RyR2, PLB phosphorylation, or NCX, Voltage-Dependent Calcium Channel (VDCC) expression [40]. In our previous study, VA vulnerability following IBR exposure demonstrated a strong correlation with reduced AMPK phosphorylation. Importantly, concurrent treatment with 5-Aminoimidazole-4-carboxamide ribonucleotide (AICAR), an AMPK activator, was associated with improvement of ventricular Ca^2+^ and voltage parameters as well as mitigation of VA vulnerability [16].

### 3.5. Possible Mechanisms Contributing to the Different Arrhythmogenic Effects of IBR and ABR

It can be speculated that the inhibition of sodium current (I_Na_), as well as the cytosolic Ca^2+^ overload, associated with BTKi may be responsible for reduced CV [33,41]. Moreover, the differential impacts of IBR and ABR as to depolarizing Na^+^ and repolarizing K^+^ current may explain the neutral effect of ABR on APD, as well as the reduced magnitude of changes in CV dispersion by ABR, as compared to IBR [41]. Although previous studies demonstrated inhibition of the PI3K-Akt pathway [11] and alterations in CSK activity [7] by IBR treatment, the above pathways were not influenced by ABR in our study. ABR, a next-generation BTKi with higher selectivity through binding to Cys-481, exhibits fewer off-target effects on other kinases and pathways than does IBR [8,42,43]. Coupled with our experimental findings, the reduced abundance of PLB in ABR-treated hearts diminishes the inhibition of SERCA2a activity [44]. These factors may underlie the distinct effects on ventricular electrophysiology and arrhythmogenesis observed between IBR and ABR. Nevertheless, further direct head-to-head clinical trials and precise mechanistic explorations, particularly those focusing on ventricular myocytes, are warranted.

### 3.6. Study Limitations

Although we have investigated the effects of IBR and ABR on ventricular conduction, repolarization, and Ca^2+^ cycling, the impacts of these agents on the individual membrane currents responsible for changes in action-potential characteristics have yet to be evaluated in our current study. However, we have summarized potential mechanisms of IBR’s association with AF based on previous experiments, which may aid in predicting the proarrhythmic potency of BTKi in the ventricle (Table S1). Nonetheless, our study provides an important insight into the electrophysiological and metabolic mechanisms behind increased VA risk associated with second-generation BTKi therapy. We acknowledge that pathophysiology is more complicated in cancer patients with co-morbidities, and our model is a healthy rodent model. Furthermore, considering the differences in ventricular ion homeostasis between species, the results from this rodent model should be translated with caution to humans, although several studies have been conducted on murine and rodent models to evaluate the cardiac electrophysiological effects of BTKi [7,12,15,16,41]. Our study used only male rats, and it is important to address sex dimorphisms in BTKi research. Our recent data suggest repolarization shortening as one of IBR’s major electrophysiological effects, and this may translate into QT shortening in a patient’s electrocardiogram (EKG). Men are associated with shorter QTc than are women, and this, we believe, contributes to sexual dimorphism in IBR-induced VA. Consistent with this concept, male patients have been shown to be more susceptible to IBR-induced cardiotoxicities, including VA [17]. In a follow-up study by Bhat et al., all of the patients with ABR-induced VA were male [13]. Both action-potential characteristics and cardiac metabolism are subjected to modulation by sex hormones. Hence, the effects may be different in male and female rats; thus, we acknowledge the limitation of including only male rats. However, given male sex as a risk factor of BTKi-induced VA, we used that fact to maximize our understanding here, and we plan to study the mechanism of the impact of sex on the arrhythmia burden in future experiments, as it is very relevant to our experiments.

## 4. Materials and Methods

The experimental protocol was approved by the University Health Network Animal Care Committee, in accordance with the rules and regulations of the Canadian Council of Animal Care (Animal Use Protocol #5969).

### 4.1. Animal Model

**Figure 5:**
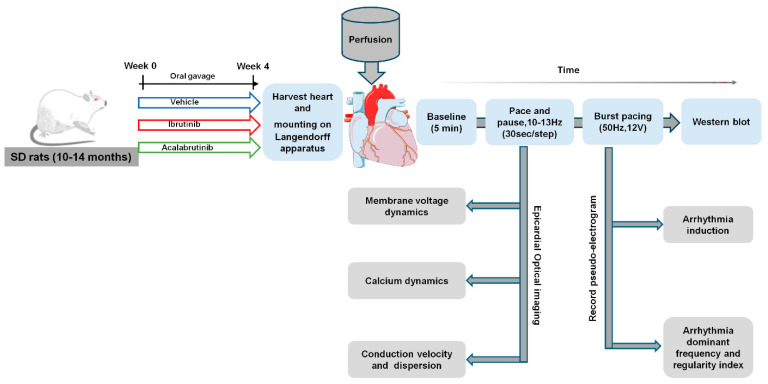
Experimental protocol and setup. Schematic diagram of the rat study protocol, groupings, treatment, and electrophysiology measurements at different steps.

A total of 30 male Sprague–Dawley (SD) rats aged 10–14 months (450 ± 50 g) were treated with an oral gavage of Ibrutinib (10 mg/day) (IBR group, *n* = 10), acalabrutinib (10 mg/day) (ABR group, *n* = 10), or an equal volume of vehicle (5% dimethyl sulfoxide) (Ctrl group, *n* = 10) for 4 weeks. Animal group sizes were as low as possible and empirically chosen. The dose of IBR was chosen based on our previous study on IBR-induced ventricular arrhythmia [16]. The dose of Acalabrutinib was primarily determined based on the dosing regimen used to evaluate its BTK inhibitory effect in two murine models of chronic lymphocytic leukemia (CLL) [45]. Subsequently, the rodent-appropriate dose was calculated using the dosage conversion relationship between rats and mice, with adjustments made for weight. Throughout the study period, the body weight of all rats was monitored every week, with any rats experiencing weight loss exceeding 10% being excluded from the follow-up experiment. There were no exclusions of animals attributed to illness/excessive weight loss during the experiments. The experimental protocol is described in Figure 5.

### 4.2. Langendorff-Perfused Rat Hearts

After 4 weeks, each rat was anesthetized with isoflurane (2–5%), and the explanted heart, after harvesting through midline thoracotomy, was cannulated and mounted to a Langendorff apparatus for retrograde perfusion with perfusate containing NaCl (130 mM), NaHCO_3_ (24 mM), KCl (4.4 mM), MgSO_4_ (0.3 mM), CaCl_2_ (2.2 mM), KH_2_PO_4_ (1.2 mM), and glucose (6 mM), and equilibrated with carbogen gas (95% O_2_ and 5% CO_2_), maintained at 37 °C, and kept under constant pressure (~70 mmHg). A pair of silver electrodes were mounted on a custom-made chair, positioned behind the heart from the camera perspective, to record pseudo-ECG.

### 4.3. Epicardial Optical Imaging

Fluorescence-based intracellular Ca^2+^ and membrane voltage dynamics were recorded simultaneously from the epicardial surface of the left ventricle with a dual optical camera. A detailed protocol for optical mapping has been previously published [15]. In brief, voltage-sensitive dye RH237 (0.25 µmol, Biotium, Inc., Fremont, CA, USA) and Ca^2+^-sensitive dye Rhod-2 AM (0.1 µmol, Biotium, Inc., Fremont, CA, USA) were slowly infused into the perfusate. Blebbistatin (6 µmol, Enzo Life Sciences, Inc., Farmingdale, NY, USA) was also added to the perfusate to suppress motion artifacts from cardiac contractions. For epicardial fluorescence recording, a xenon light source (Moritek, Saitama, Japan) and a 530 nm green filter (Semrock, Rochester, NY, USA) were utilized to illuminate the left ventricle and excite dye fluorescence. A dichroic mirror at 665 nm divided the emission light beam. Light wavelengths above 665 nm were filtered through a pass with a length of 715 nm for action potential (AP) measurement, while those below 665 nm were filtered through a 585/40 nm bandpass filter for the measurement of Ca^2+^ transients. Voltage and Ca^2+^ signals were simultaneously recorded using different high-speed CMOS cameras (Ultima-L, Scimedia, Costa Mesa, CA, USA), each equipped with 10,000 pixels arranged in a 100 × 100 matrix on a 1-cm^2^ sensor, and operating at a frequency of 500 frames per second. The optical arrangement included a Leica Plan APO 0.63× lens at the objective and a 1.0× on the condensing side, resulting in a spatial resolution of 160 µm/pixel. Electrical stimulation of the ventricle was delivered by a Grass Instruments S88X pulse stimulator through a pair of silver electrodes 1 mm apart connected to the epicardial surface of the ventricle; the electrodes were mounted on a homemade support made of syringe needles. Voltage and Ca^2+^ parameters were recorded with incremental pacing according to a “pace-&-pause” protocol [15]. Hearts were paced for 30 s with burst pacing in a frequency range from 10.0 to 13.0 Hz to achieve a steady-state (6 V output, pulse width of 4 ms), and AP and Ca^2+^ signals were recorded during the last 2 s of pacing and the first 2 s of post-pacing intrinsic rhythm.

Voltage and Ca^2+^ signals obtained from optical mapping were imported into MATLAB for subsequent signal processing and calculation of AP and CaT parameters. APD was measured from the onset of AP to 80% of repolarization (APD_80_). Beat-to-beat variation of APD_80_ (APD alternans) was used as a measure of the dispersion of repolarization. APD spatially discordant alternans was quantified as the width of the half-peak value of the APD alternations distribution histogram. The ΔAPD represented the average APD from even and odd beats. CaT rise time was calculated as the time required for fluorescence to rise from 10% to 90% during systolic release [46]. CaT decay time and spontaneous calcium elevation (SCaE) were assessed as markers of the diastolic Ca^2+^ load [46,47]. CaT amplitude ratio was calculated by dividing the smallest Ca^2+^ signals by the largest Ca^2+^ signals. A smaller ratio represents higher Ca^2+^ alternans, and vice versa [47].

Other than AP and CaT parameters, CV and CV dispersion were also measured. CV was determined by analyzing the propagation of the voltage signal during electrical stimulation using optical mapping data, and isochrone maps were constructed for each stimulated heartbeat, with the maximum positive time derivative serving as the reference point for activation time. Each map was divided into a 10 × 10 grid, and CV estimation was computed in the middle of each of the 81 squares constituting this grid. For each square, the activation time measured at the 4 corners was fitted into a polynomial surface, and CV was computed using a method similar to that used by Bayly and colleagues [48], giving, for each map, a 9 × 9 CV vector field. The CV dispersion at each stimulation frequency was calculated by dividing the standard deviation of CV at each point in the mapping area by the mean CV [30].

### 4.4. Determination of Ventricular Arrhythmia Vulnerability and Characteristics

At the end of optical mapping, hearts were subjected five episodes of VA induction by direct electrical stimulation with burst-pacing at 50 Hz and 12 V for 10 sec, with an interval of 2–3 min for recovery between inductions [15]. All VA episodes induced by electrical stimulation and persisting for ≥10 s were considered to be successfully induced VA and were included in the analyses of VA vulnerability and VA burden, as well as the calculation of VA dominant frequency (DF) and the regularity index (RI). Successful induction of VA was calculated from the percentage of successfully induced VF episodes in each heart during five inductions and was used as a measure of VF inducibility [15]. The mean duration of successfully induced VA (in seconds) for each heart was used as a parameter to assess the VA burden. The ECG signals of all VAs ≥ 10 s were subjected to power spectral density (PSD) analysis between 0–20 Hz. The frequency associated with the highest energy component was extracted as the DF [49,50]. The VA phenotypes were classified according to methods described in the previous literature [20]. The ratio of the power at the DF and the adjacent 1 Hz width to the total power (the sums over the range of 0–20 Hz) was defined as RI, an effective method used to directly quantify temporal organization [49,50]. The RI values range from 0 to 1, where higher values denote organized VA and lower values signify disorganized VA. We also performed optical mapping during the sustained VAs (VA ≥ 60 s). An analysis of the phase mapping of voltage recordings was conducted as a surrogate for VA spatial organization, similarly to that described in our previously published methodology. The analysis was performed on the total count of waves from voltage and calcium data observed during the two 10 s segments of VA [51,52].

### 4.5. Immunoblotting

Proteins extracted from the stored LV tissue were analyzed with standard Western blot protocols [15]. Equal amounts of proteins were separated on SDS-PAGE, transferred to PVDF membranes, and probed with the following primary antibodies: total and phospho-PLB (Badrilla), SERCA2a (Badrilla), NCX (biorbyt), phospho-RyR2 (Badrilla), total RyR2 (Thermo Fisher), CaMKII (pThr286 or 287) (Badrilla), CaMKII delta (abcam), total and phospho-AMPK(pThr172) (Cell signaling), GAPDH (Fitzgerald), and horseradish peroxidase-conjugated goat anti-rabbit or rabbit anti-mouse secondary antibodies. The proteins were detected with Image Lab Touch Software (version 2.4.0.03, Chemidoc Imaging System from Bio-Rad, Hercules, CA, USA), and Western blot bands were quantified by Image J (version 2.1.0); the results are presented in bar graphs.

### 4.6. Statistical Analysis

Experimental data are expressed as mean± standard error of the mean. Data distribution was evaluated by the Shapiro–Wilk normality test. Student’s *t*-test was performed to compare VA dominant frequency and VA regularity index between the IBR and ABR groups. Arrhythmia morphologies (monomorphic vs. polymorphic) were analyzed by Χ^2^ test. Two-way repeated-measures ANOVA was performed for continuous variables in the analysis of APD_80_, APD alternans, CV, CV dispersion, CaT rise time, CaT decay time, SCaE, and CaT ratio. One-way ANOVA (for normally distributed data with equal variance) or the Kruskal–Wallis test (for non-normally distributed data) were performed to compare the differences between the three groups. Statistical significance was defined as *p* < 0.05. Data analysis was performed using the Prism 9.00 (GraphPad, San Diego, CA, USA) software.

## 5. Conclusions

As previously observed with Ibrutinib, chronic Acalabrutinib treatment also resulted in increased risk of ventricular arrhythmia in our rodent model. Although reduced AMPK phosphorylation and abnormal Ca^2+^ dynamics are common to both generations of BTKi, the differential impacts of these agents on membrane electrophysiology may explain the relatively lower risk and more spatially organized ventricular arrhythmia following Acalabrutinib treatment, compared to Ibrutinib. In our model, a more organized ventricular arrhythmia with Acalabrutinib may particularly result from its neutral effect on repolarization and repolarization alternans. However, further clinical studies are necessary to determine whether Acalabrutinib will lead to more monomorphic ventricular tachycardia in humans. Additionally, there might be differences in responses to various therapies associated with Acalabrutinib-induced and Ibrutinib-induced ventricular arrhythmias.

## Data Availability

The data presented in this study are available on request from the corresponding author.

## Supplementary Materials

The following supporting information can be downloaded at: https://www.mdpi.com/article/10.3390/ijms25116207/s1.
