# Supplementary material for: Arrhythmogenic Ventricular Remodeling by Next-Generation Bruton’s Tyrosine Kinase Inhibitor Acalabrutinib
Source: Int J Mol Sci. 2024 Jun 5;25(11):6207. doi: 10.3390/ijms25116207 (PMC11173147; doi:10.3390/ijms25116207)
Supplement: Supplementary file 1 [file ijms-25-06207-s001.zip › Figure S1,S2.docx]

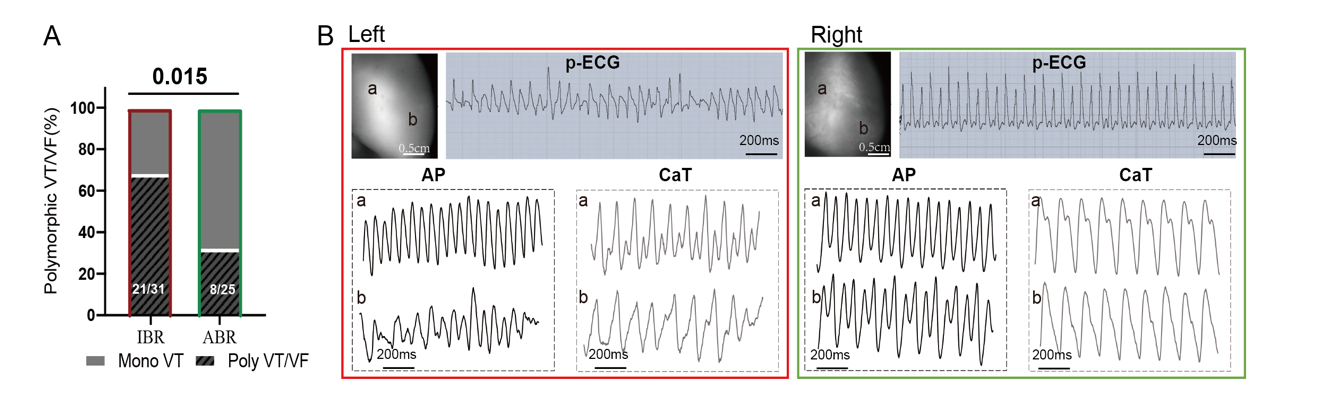


**Figure S1. Phenotype of IBR and ABR induced VA.** (A) Percentage of polymorphic ventricular tachycardia (VT)/VF and monomorphic VT in IBR and ABR groups. The total times of each group with polymorphic VT/VF and the total number of ventricular arrhythmia (VA) induction are presented within the associated bars. (B) Typical examples of pseudo-electrogram(p-ECG), corresponding action potential(AP), and calcium transient(CaT) recording during VT in Ibrutinib (IBR; Left) and Acalabrutinib (ABR; Right) heart. Representative the optical signal acquisition areas of the right ventricle(a) and left ventricle (b) respectively. (p *value*: x^2^ test).


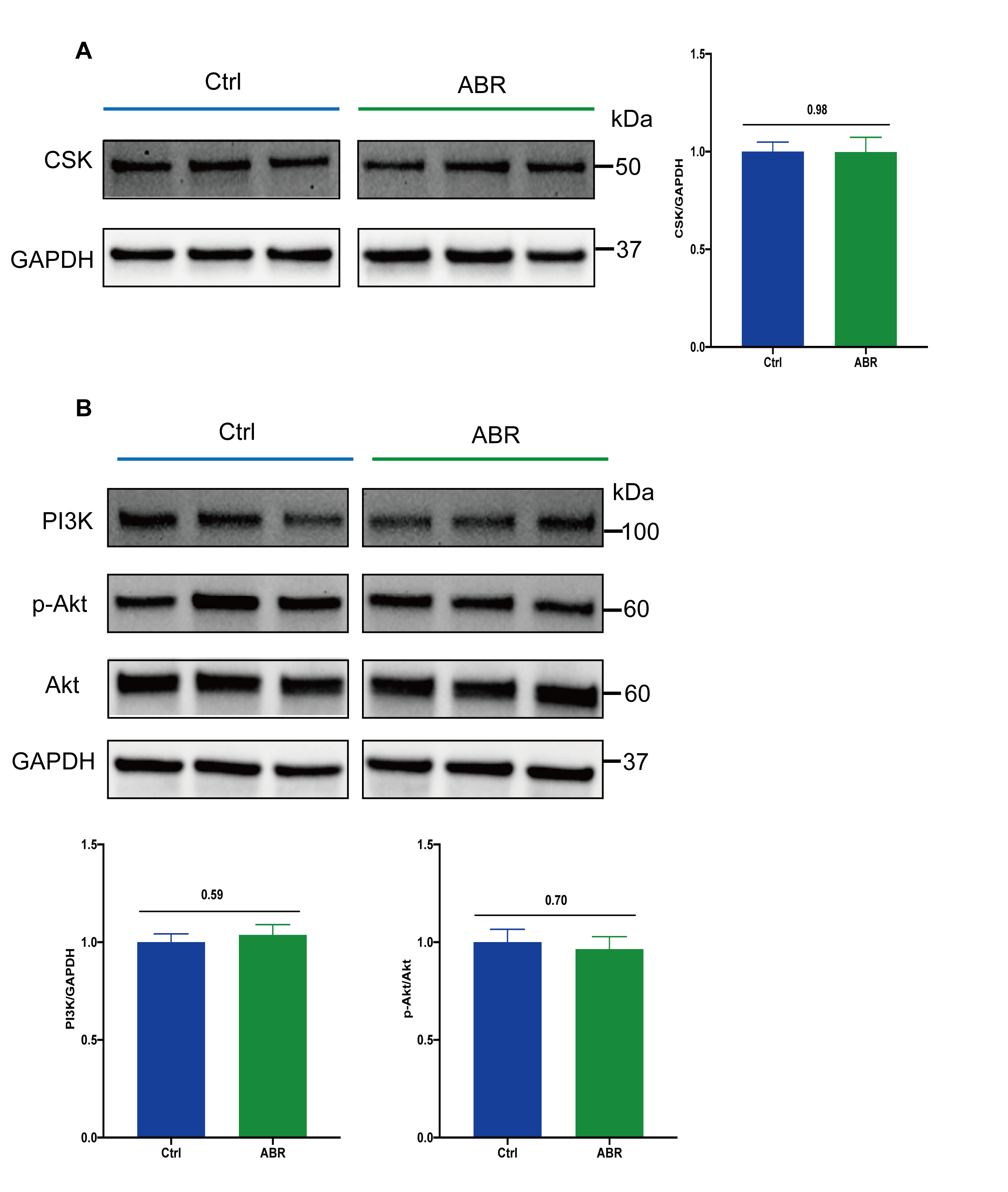


**Figure S2. Effect of ABR on CSK and PI3K/Akt pathway.** (A) Representative gel blots and normalized the expression of C-terminal Src kinase (CSK). (B)Representative gel blots and normalized the expression of phosphoinositol-3 kinase (PI3K), phosphorylation of Akt. (n=10 in each group; p *value*: t- test). ABR- Acalabrutinib; Ctrl-control; GAPDH- glyceraldehyde 3-phosphate dehydrogenase.
