# Supplementary material for: Arrhythmogenic Ventricular Remodeling by Next-Generation Bruton’s Tyrosine Kinase Inhibitor Acalabrutinib
Source: Int J Mol Sci. 2024 Jun 5;25(11):6207. doi: 10.3390/ijms25116207 (PMC11173147; doi:10.3390/ijms25116207)
Supplement: Supplementary file 1 [file ijms-25-06207-s001.zip › ijms Table S1.docx]

Table S1. Summary of research on the proarrhythmic potency of the BTKi drugs. ABR, acalabrutinib; AF: atrial fibrillation; APD, action potential duration; AT: atrial tachycardia; Ca^2+^, calcium; CaT, calcium transient; CaTD50,the duration from depolarization to 50% of repolarization of CaTs; CSK, C-terminal Src Kinase; CV, conduction velocity; IBR, ibrutinib; I_Kur_, ultrarapid delayed rectifier K^+^ current; I_to_, transient outward K^+^; NC; unclear; NR, unresearched; NRVM, neonatal rat ventricular myocytes; SCaR, spontaneous calcium release; SR, sarcoplasmic.

| **BTKi** | **Model** | **Administration mode and dosage** | **Mechanisms** | **Effect on electrophysiology** | **Comment** | **Refs** |
| --- | --- | --- | --- | --- | --- | --- |
| IBR | NRVM | In-vitro; 0.01~1μM | Downregulate PI3K-Akt | NR | Increased incidence of AF | [1] |
| IBR | C57BL/6 Mice | In vivo;10mg/kg; acute | NR | NR | Increased incidence of AT/AF | [2] |
|  |  | In vivo;10mg/kg; chronic(14 days)-washout(24hours);  In vivo;2mg/kg; acute | NR | NR | No impact on the incidence of AT/AF |  |
| IBR | C57BL/6 Mice | In vivo ;25mg/kg/d; chronic (4 weeks) | Increased CaMKII;  Increased RyR2-Ser2814;  Increased PLN-Thr17 | Decreased CaT amplitude;  Increased SCaR;  Decreased SR Ca^2+^ capacity | Increased incidence of AF | [3] |
| IBR | Rat | Ex-vivo; 0.1μM | NC | Increased Ca^2+^ alternans;  Increased APD alternans;  Reduced CaTD50;  Slower AP upstroke velocity | Increased incidence of VF | [4] |
| IBR | C57BL/6 Mice | In vivo; 25mg/kg/d; chronic (4 weeks) | Inhibit CSK activity | NR | Increased incidence of VF | [5] |
| ABR |  | In vivo; 25mg/kg/d; chronic (4 weeks) | No effect on CSK activity | NR | No impact on the incidence of AF |  |
| **BTKi** | **Model** | **Administration mode and dosage** | **Mechanisms** | **Effect on electrophysiology** | **Comment** | **Refs** |
| ABR | C57BL/6 Mice | In vivo;10mg/kg; acute | NR | No effect on cardiac electrophysiology | No impact on the incidence of AF | [6] |
|  | Isolated atrial | Ex-vivo; 10μM | NR |  | NR |  |
|  | Isolated atrial | Ex-vivo; 50μM | Block I_Kur_ | Prolong ADP | NR |  |
| IBR | Isolated atrial | Ex-vivo; 10μM | Reduced I_Na_ density; Block I_Kur_ and I_to_ | Decreased CV; Prolong ADP | Increased incidence of VF |  |

1. McMullen, J. R.; Boey, E. J.; Ooi, J. Y.; Seymour, J. F.; Keating, M. J.; Tam, C. S. Ibrutinib increases the risk of atrial fibrillation, potentially through inhibition of cardiac PI3K-Akt signaling. *Blood* **2014,** 124, (25), 3829-3830.

2. Tuomi, J. M.; Xenocostas, A.; Jones, D. L. Increased Susceptibility for Atrial and Ventricular Cardiac Arrhythmias in Mice Treated With a Single High Dose of Ibrutinib. *The Canadian journal of cardiology* **2018,** 34, (3), 337-341.

3. Jiang, L.; Li, L.; Ruan, Y.; Zuo, S.; Wu, X.; Zhao, Q.; Xing, Y.; Zhao, X.; Xia, S.; Bai, R.; et al. Ibrutinib promotes atrial fibrillation by inducing structural remodeling and calcium dysregulation in the atrium. *Heart rhythm* **2019,** 16, (9), 1374-1382.

4. Du, B.; Chakraborty, P.; Azam, M. A.; Massé, S.; Lai, P. F. H.; Niri, A.; Si, D.; Thavendiranathan, P.; Abdel-Qadir, H.; Billia, F.; et al. Acute Effects of Ibrutinib on Ventricular Arrhythmia in Spontaneously Hypertensive Rats. *JACC. CardioOncology* **2020,** 2, (4), 614-629.

5. Xiao, L.; Salem, J. E.; Clauss, S.; Hanley, A.; Bapat, A.; Hulsmans, M.; Iwamoto, Y.; Wojtkiewicz, G.; Cetinbas, M.; Schloss, M. J.; et al. Ibrutinib-Mediated Atrial Fibrillation Attributable to Inhibition of C-Terminal Src Kinase. *Circulation* **2020,** 142, (25), 2443-2455.

6. Tuomi, J. M.; Bohne, L. J.; Dorey, T. W.; Jansen, H. J.; Liu, Y.; Jones, D. L.; Rose, R. A. Distinct Effects of Ibrutinib and Acalabrutinib on Mouse Atrial and Sinoatrial Node Electrophysiology and Arrhythmogenesis. *Journal of the American Heart Association* **2021,** 10, (22), e022369.
