# Supplementary material for: Arrhythmogenic Ventricular Remodeling by Next-Generation Bruton’s Tyrosine Kinase Inhibitor Acalabrutinib
Source: Int J Mol Sci. 2024 Jun 5;25(11):6207. doi: 10.3390/ijms25116207 (PMC11173147; doi:10.3390/ijms25116207)
Supplement: Supplementary file 1 [file ijms-25-06207-s001.zip › Video Caption.docx]

Video S1: Phase maps of Ibrutinib heart during VA

Video S2: Phase maps of Acalabrutinib heart during VA
